# Natural Products for Antithrombosis

**DOI:** 10.1155/2015/876426

**Published:** 2015-05-17

**Authors:** Cen Chen, Feng-Qing Yang, Qian Zhang, Feng-Qin Wang, Yuan-Jia Hu, Zhi-Ning Xia

**Affiliations:** ^1^School of Chemistry and Chemical Engineering, Chongqing University, Chongqing 400030, China; ^2^State Key Laboratory of Quality Research in Chinese Medicine, Institute of Chinese Medical Sciences, University of Macau, Macau

## Abstract

Thrombosis is considered to be closely related to several diseases such as atherosclerosis, ischemic heart disease and stroke, as well as rheumatoid arthritis, hyperuricemia, and various inflammatory conditions. More and more studies have been focused on understanding the mechanism of molecular and cellular basis of thrombus formation as well as preventing thrombosis for the treatment of thrombotic diseases. In reality, there is considerable interest in the role of natural products and their bioactive components in the prevention and treatment of thrombosis related disorders. This paper briefly describes the mechanisms of thrombus formation on three aspects, including coagulation system, platelet activation, and aggregation, and change of blood flow conditions. Furthermore, the natural products for antithrombosis by anticoagulation, antiplatelet aggregation, and fibrinolysis were summarized, respectively.

## 1. Introduction

The hemostatic system, which comprises platelet aggregation, coagulation, and fibrinolysis, is a host defense mechanism that preserves the integrity of the high pressure closed circulatory system in mammals after vascular damages [1]. Under normal physiological conditions, the thrombi formation, controlled by the regulatory system, is temporary and spatial [2–5]. However, when pathological processes overwhelm the regulatory system of hemostasis or a shift in the hemostatic balance towards the procoagulant side, thrombosis is initiated [6]. Under this hypercoagulable state, excessive quantities of thrombi will be formed, which will ultimately lead to parts or total blockage of blood vessels [7, 8]. The development of clots in the artery, vein as well as microvascular circulation is the most frequent cause of morbidity and mortality worldwide [9, 10]. The formation of thrombi in the arterial circulation usually occurs in individuals at high risk of cardiovascular diseases [11] and coronary myocardial infarction and ischemic stroke are the main results of atherosclerosis and thrombosis in the coronary arteries [12]. Furthermore, peripheral arterial diseases including mesenteric artery embolism and limb arterial thrombosis are also closely related to the arterial thrombosis. Venous thromboembolism (VTE), consisting of deep vein thrombosis (DVT) and its complication, pulmonary embolism (PE), is a relatively common condition that associated with serious symptoms [13, 14]. In reality, venous thrombosis is the second leading cause of death in patients with cancer. In addition, disseminated intravascular coagulation and microangiopathy hemolytic anemia (thrombotic thrombocytopenic purpura (TTP) and hemolytic uremic syndrome (HUS)) are associated with microvascular thrombotic disorders [6]. Therefore, more and more studies have been focused on preventing thrombosis for the treatment of those thrombotic diseases.

In recent years, antithrombotic drugs, which can be classified into three major categories including anticoagulation, antiplatelet aggregation, and fibrinolysis, have been intensively studied and developed as potential therapeutic approaches for arterial and venous thrombosis [15, 16]. Among these clinical used drugs, heparin [17], warfarin [18], and their derivates are mainly applied in inhibition of the blood coagulation factors, while plenty of antiplatelet drugs such as aspirin (ASP), clopidogrel, and abciximab have been used in reducing the risk of cardiovascular diseases [19–22]. Furthermore, fibrinolytic agents, such as streptokinase, tissue plasminogen activator (t-PA), and reteplase, are engaged to remove and dissolve the formed blood clots [23, 24]. Despite intense investigation over the last 40 years into the discovery and development of more effective antithrombotic drugs, the effect of these therapies on mortality rates still remained small [25]. And this situation will probably become more challenging in the future as the incidences of obesity, diabetes, and the metabolic syndromes rapidly increase. The reasons of low cure rates of these drugs mainly lie in drug resistance, limited efficacy in some patients, and side effects such as higher bleeding risk and gastrointestinal dysfunctions [26]. A study in United Kingdom, researchers indicated that the responsible drug for over 60% of the deaths caused by adverse drug reactions is ASP [27]. The side effects of ASP include bleeding, gastrointestinal toxicity, and thrombocytopenia. Cilostazol, a potent inhibitor of cyclic adenosine monophosphate- (cAMP-) phosphodiesterase 3 (PDE_3_), has serious side effects such as headache and palpitation [28]. Apixaban is an oral selective direct factor Xa (FXa) inhibitor and its most common adverse event is bleeding [29], and other adverse events reported are hypersensitivity reactions, syncope, nausea, dizziness, and so forth. Therefore, there is a rising urgent need for novel therapeutic approach to reduce current adverse effects of antithrombotic drugs without impairing their efficacy.

Nowadays, much effort has been focused on the discovering of natural products as effective supplements or even substitutes to those currently used antithrombotic drugs [30]. These natural products, composing of natural plants [31–33], traditional Chinese medicines (TCMs) [34, 35], and functional foods [36–38] as well as some special animal materials [39], have been found to possess remarkable antithrombotic property both in experimental and clinical stages. It is known to all that TCMs have a long history for treating many kinds of human diseases including thrombotic diseases and blood stasis syndromes. In reality, in Shennong's Classic of Materia Medica (Shennong Bencao Jing in Chinese) [40], 83 of 365 TCMs were recorded with the function of “HuoXueHuaYu,” which means to promote blood circulation for removing blood stasis. Nowadays, there are some natural products that have been used in clinic for the treatment of thrombotic diseases. For example, Shimotsu-To, which is a combined prescription of four herbal extracts,* Paeonia lactiflora*,* Rehmannia glutinosa*,* Angelica sinensis*, and* Ligusticum chuanxiong*, has been used in clinic for improving abnormal blood coagulation, fibrinolysis, and atherosclerosis [41]. Kang naoxueshuan (in Chinese) tablet, which consists of* Flos Carthami*,* Radix Angelicae Sinensis*,* Hirudo*, and so forth, can protect cerebral ischemia through antiplatelet aggregation and reduction of blood viscosity [42]. Besides,* Ginkgo biloba* leaves tablets are widely used in treating ischemic cerebrovascular diseases [43]. The main reasons for applying natural products to the treatment of thrombotic diseases are that they comprise multiple constituents and each constituent may have multiple targets; they may exert pleiotropic and synergistic effects that have positive functions for increasing the therapeutic efficacy. Besides, the constituents of natural products usually have less side effects on the gastrointestinal system [44].

This review will provide an overview on the formation mechanisms of thrombosis and the antithrombotic properties exerted by natural products and describe the pathways by which their activities may contribute to reduce thrombotic risks.

## 2. The Formation of Thrombosis

Thrombus can be classified into four groups based on different positions and constituents [45]: (1) pale thrombus, mainly occurs in fast-flowing blood with numerous platelets; (2) red thrombus, constituting of fibrin and erythrocyte in slow-flowing blood; (3) mixed thrombus, a continuous process of thrombus formation; (4) hyaline thrombus (also called microthrombus), the formation of cellulose in microcirculation small vessels. On the other hand, venous thrombosis, arterial thrombosis, and microvascular thrombosis are more likely to be distinguished depending on different blood vascular systems [46].

Thrombus formation, including platelet adhesion, activation, secretion, and aggregation as well as tissue factor (TF) initiating thrombin generation and fibrin formation, is highly complex [1]. When the vessel wall is breached or the endothelium is disrupted, collagen, and TF become exposed to the flowing blood, thereby initiating formation of a thrombus. Exposed collagen triggers the accumulation and activation of platelets, whereas exposed TF initiates the generation of thrombin, which not only converts fibrinogen to fibrin but also further activates platelets [8]. In this paper, the formation of thrombi is described in brief on three aspects, including coagulation system, platelet activation, and aggregation, and the change of blood flow conditions.

### 2.1. Coagulation System

**Figure 1:**
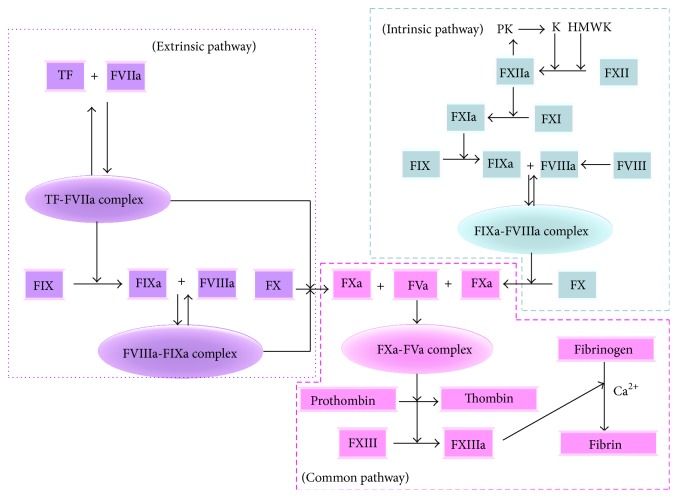
Extrinsic, intrinsic, and common pathways of blood coagulation during hemostasis and thrombosis. PK: prekallikrein; K: kallikrein; HMWK: high molecular weight kininogen.

Blood coagulation and platelet adhesion and activation are critical for cessation of blood loss at sites of vascular injury in the high-pressure closed circulatory system [47]. Upon vessel injury, coagulation system can be activated via either the contact activation (or intrinsic) pathway or by the TF (or extrinsic) pathway and converge on a common (intrinsic + extrinsic) pathway, which starts at the level of factor X (FX) to lead to thrombin and fibrin formation [48]. The extrinsic pathway is initiated by excessive exposure of TF which is a 263-residue membrane-bound glycoprotein [49] and as receptor and cofactor for factor VII (FVII) and its active form VIIa (FVIIa) [3, 50, 51]. On binding of FVIIa to TF, complex (TF-FVIIa) acquires catalytic activity and converts factors IX (FIX) and X (FX) to their active derivatives factors IXa (FIXa) and Xa (FXa), respectively [52]. Simultaneously, the intrinsic pathway begins with formation of the primary complex on collagen by high-molecular-weight kininogen, prekallikrein, and FXII. FXII firstly becomes FXIIa; and FXIIa converts FXI to FXIa. FXIa activates FIX, which with its cofactor FVIIIa forms the tenase complex and then activates FX to Fxa [53]. In the common pathway, FXa derived from both intrinsic and extrinsic processes with FVa on membrane surface in complex with prothrombinase complex activates thrombin formation which finally converts fibrinogen to fibrin polymers [54, 55] (Figure 1).

### 2.2. Platelet Activation and Aggregation

The intact vascular endothelium is a semipermeable barrier that controls the diffusion of plasma molecules, regulates vascular tone and inflammatory, and releases gaseous signal molecule including nitric oxide (NO) and prostacyclin (PGI_2_) as well as endothelial CD_39_ to prevent platelet aggregation or dilate blood vessels under physiological conditions. However, dysfunctional or impaired endothelium is characterized by the loss of such antiplatelet properties and tends to mediate and accelerate thrombosis. The exposure binding sites of collagen and von Willebrand factor (vWF), a multimeric plasma glycoprotein, allow the platelet membrane glycoprotein (GPIb-IX-V or GPVI) to adhere on it in the first place. After the initial adhesion of platelets to the extracellular matrix, platelets undergo shape change and the activation process requires a rapid response to autocrine and paracrine mediators, including adenosine diphosphate (ADP), thrombin (THR), epinephrine, and thromboxane A2 (TXA_2_) [56]. Furthermore, platelet granule secretions lead to the local release of ADP/adenosine triphosphate (ATP), 5-hydroxytryptamine (5-HT), Ca^2+^, adhesion proteins (e.g., fibrinogen, fibronectin, thrombospondin, vitronectin, P-selectin, and GPIIb/IIIa), and coagulation factors (factor V,  factor XI, plasminogen activator inhibitor type 1, plasminogen, and protein S), all of which contribute to perpetuate and amplify the thrombotic response [57]. These platelet agonists binding to specific membrane receptors (e.g., collagen binds to GPVI or *α*_2_ *β*_1_, THR interacts with protease activated receptors, and ADP binds at least two ADP receptors on platelets) [58–60] activate phospholipase C*β* (PLC*β*), resulting in the production of diacylglycerol (DAG) and inositol trisphosphate (IP_3_). DAG and IP_3_ activate protein kinase C (PKC) and mobilize cytoplasmic Ca^2+^, respectively. Then TXA_2_ is produced as a consequence of increased cytoplasm Ca^2+^-levels and the high concentration of Ca^2+^ is necessary for the activation of PLA2 through phosphorylation by p-38-mitogen-activated protein kinase (MAPK) [61]. Platelet aggregation is regulated in the final part of the pathway by activation of the platelet heterodimer GPIIb/IIIa receptor, the most abundant proteins on the platelet surfaces. Fibrinogen, the main ligand for the GPIIb/IIIa receptor, binding to GPIIb/IIIa also triggers an “inside out” signaling, causing amplification of the initial signal and further platelet activation. In the final phase of thrombus formation, fibrinogen is converted to fibrin by thrombin, leading to the stabilization of the platelet aggregates with more platelets and blood cells (leukocytes and red blood cells), thus getting trapped and contributing to growth of thrombus [62].

### 2.3. Change of Blood Flow Conditions

Physiologically, plasma separates blood vessel from the tangible components such as erythrocyte, leukocyte, and platelet in blood. Once the blood flow slows down, platelet will move to the edge of blood vessel as well as adhere to the impaired endometrial, coagulator factors will be activated, and thrombin accumulates and amounts to a high concentration to facilitate thrombus formation. Furthermore, the blood viscosity [63], which will result in a lower erythrocytic deformability and a stronger platelet aggregation, will increase under slow blood flow condition. This cycling process between increasing erythrocytic deformability and slowing down blood flow finally promotes the adherence and aggregation of platelet. As a result, it is easy to form thrombus in vein with slow blood flow, where the concentration of coagulation factors and thrombin are very high locally [64, 65]. On the contrary, in artery where coagulation factors and thrombin can be scattered by fleet blood flow and it is less likely to achieve effective concentrations, so the thrombus formation in artery mainly relies on the adherence, activation, and aggregation of platelet rather than the impacts of coagulation factors and thrombin [66].

## 3. Antithrombotic Effects of Natural Products

Studies have demonstrated that natural products become increasingly crucial in reducing the thrombotic risks and treating various cardiovascular diseases. As previously mentioned, drugs for treating thrombosis can be divided into three categories: (1) anticoagulants, which prevent the coagulation system and interfere with further plaque expansion; (2) antiplatelet agents, which decrease platelet aggregation and inhibit thrombus formation; (3) fibrinolytic drugs, which dissolve the formed thrombus directly [67].

### 3.1. Anticoagulation

The extrinsic and intrinsic coagulation systems are initiated after vascular disruption via TF and collagen, respectively [8]. In clinical treatment, inhibition of coagulation system is an effective way to prevent the pathological thrombus formation.

#### 3.1.1. Inhibition of Tissue Factors

TF as a membrane protein and the main initiator of the coagulation cascade is essential for thrombus formation [68]. TF expression in endothelial cells is induced by different inflammatory mediators including tumor necrosis factor- (TNF-) *α* [69], interleukin- (IL-) 1*β* [70], or histamine [71]. In reality, reducing TF expression significantly impairs thrombus formation, and agents focused on inhibition of TF activation become increasingly used effective clinical methods to treat coagulation diseases.

It has been reported that* Chaenomeles sinensis* has antithrombotic and antiplatelet aggregation activities [72]. Thirteen components were isolated and purified from the fruits of* C. sinensis* and five of them including hovertrichoside C (IC_50_ = 14.0 *μ*g), luteolin-7-O-*β*-D-glucuronide (IC_50_ = 31.9 *μ*g), hyperin (IC_50_ = 20.8 *μ*g), avicularin (IC_50_ = 54.8 *μ*g) and quercitrin (IC_50_ = 135.7 *μ*g) can inhibit the TF expression of rat plasma after the addition of CaCl_2_ *in vitro*. Furthermore, the TF inhibitory activity of the C-ring pentacyclic flavonol was evidently stronger than that of C-ring hexacyclic flavonol [73]. Rhizoma Ligustici Chuanxiong (with the main active component ligustrazine) is widely used in treating cardiovascular diseases, pulmonary hypertension, chronic renal failure and liver cirrhosis [74]. Shang et al. reported the inhibitory effects of ligustrazine on the expression of TF and vWF in human blood induced by THR* in vitro*. The result showed that ligustrazine suppressed TF expression not only in quiescent condition but after being induced by THR, and also decreased vWF formation after being induced by THR. These results provide a scientific basis for Rhizoma Ligustici Chuanxiong to be used as an antithrombotic agent [75]. In addition, a sesquiterpene glycoside (3-O-*α*-L-rhamnopyranosyl-(→4)-*α*-L-rhamnopyranosyl-(1→2)-*α*-L-(4-trans-feruloyl)-rhamnopyranosyl-(1→6)-*β*-D-glucopyranosyl) isolated from the leaves of* Eriobotrya japonica *Lindley (Rosaceae) showed a strong TF inhibitory activity (IC_50_ = 2 *μ*M)* in vitro* and another component ferulic acid illustrated a weak inhibitory activity (IC_50_ = 369 *μ*M). This active sesquiterpene glycoside was composed of three parts including nerolidol, carbohydrate and feruloyl moieties, and the nerolidol moiety was mainly responsible for the inhibitory effect against TF [76].

In addition, estrogen replacement therapy could protect cardiovascular system and decrease the incidence of related diseases [77]. *α*-Zearalanol (ZAL), which is one of the natural phytoestrogens usually found in beans and grain, could decrease the contents of TF and its expression on vascular endothelium in rat plasma* ex vivo* with similar to or better than that of positive drug 17*β*-estradiol [78].

#### 3.1.2. Inhibition of the Coagulation Pathways

The pathways of the coagulation system mainly consist of two distinct cascades (intrinsic and extrinsic coagulation pathways) ultimately contributes to the formation of the key protease thrombin which in turn converts fibrinogen into fibrin to stabilize the formed platelet-rich plug. In experiment models, activated partial thromboplastin time (APTT), prothrombin time (PT) and thrombin time (TT) are tested to indicate the activation of intrinsic, extrinsic and their common (intrinsic + extrinsic) pathway, respectively [79]. The anticoagulation effects by inhibition of the coagulation pathways of natural products are summarized in Table 1.

The green algae* Monostroma arcticum* (MA), with polysaccharide as its important bioactive substance, is widely distributed in China. A polysaccharide HAF0 (average molecular weight of 9.36 kDa) isolated from MA showed the inhibition effect on the intrinsic and/or common coagulation pathway with prolonging APTT and TT [80].* Polygala fallax* Hesml. (PFH) is used as a folk medicine for antiaging, preventing myocardial ischemia and regulation of immune system. The anticoagulation and antithrombotic effects of the total saponins from PFH was mainly contributed to the inhibition of intrinsic coagulation system by prolonging APTT, plasma recalcification time (RT) as well as THR-induced fibrinogen clotting time, but did not impact on PT [81]. In reality, the anticoagulation mechanisms for most of the drugs mainly rely on inhibition of both intrinsic and extrinsic, or common coagulation pathways. Hyperoside, isolated from the leaves of* Rhododendron brachycarpum*, was observed* ex vivo *in mice with dose-dependent prolongation of the APTT and PT as well as inhibited platelet aggregation induced by THR and collagen* in vitro*, ADP* in vivo* [82]. Polysaccharide from* Umbilicaria esculenta* inhibited the thrombus formation in a dose-dependent manner using an arteriovenous shunt thrombosis model in rats, and the more prolongation of APTT suggested a more obvious inhibition of the intrinsic than the extrinsic coagulation systems [83]. Withaferin A (WFA), an active compound from* Withania somnifera*, is widely studied on its effects on inflammatory, cardiovascular and central nervous system [84]. It is reported that WFA significantly prolonged APTT as well as PT, inhibited the activities and production of thrombin and FXa following extending* in vivo* and* ex vivo* bleeding time, and inhibited the production of TNF-*α* induced plasminogen activator inhibitor type 1 (PAI-1), an important component of the coagulation system that down-regulates fibrinolysis in the circulation [85]. Those results indicated that WFA possessed antithrombotic activities and might be developed as a new anticoagulant agent [86]. Wogonin (WGN) as well as its metabolite wogonoside (WGNS) is the flavonoids from* Scutellaria baicalensis* Georgi [87]. Treatment with WGN and WGNS resulted in prolonging APTT and PT as well as inhibition of the activities and production of THR and FXa in tumor necrosis factor- (TNF-) *α* activated human umbilical vein endothelial cells [88]. Pawlaczyk et al. studied the anticoagulant and antiplatelet activities of different fractions of* Erigeron canadensis *L. The mixture parts of polysaccharide-polyphenolic macromolecules inhibited both intrinsic and extrinsic coagulation pathways, as well as platelet aggregation induced by collagen* in vitro*. While in the carbohydrate part, only glucuronic acid and galacturonic acid showed weak anticoagulant activity [89]. In addition, the anticoagulant effect of total glycosides of paeony included prolonging APTT, PT, and TT* in vitro* confirmed that intrinsic, extrinsic, and common coagulation pathways were all inhibited [90].

### 3.2. Anti-Platelet Aggregation

The inhibition of platelet function has been widely studied for a long time in an effort to prevent and treat thrombosis, especially in antiplatelet aggregation. Andrographolide, the active component of* Andrographis paniculata*, could inhibit PAF-induced human blood platelet aggregation in a dose-dependent manner (IC_50_ ≈ 2 *μ*M) [91]. Bupleurumin from the aerial parts of* Bupleurum falcatum* showed an 8-fold potent inhibitory effect (IC_50_ = 47.5 *μ*M) compared to that of ASP (IC_50_ = 420 *μ*M) on collagen-induced platelet aggregation, and comparable inhibitory effects as ASP on AA-induced platelet aggregation [92]. In Maione's study, Tanshinone IIA (TIIA) selectively inhibited rat platelet aggregation induced by reversible ADP stimuli (3 *μ*M) in a concentration-dependent manner (0.5–5 *μ*M). Nevertheless, TIIA was less active against the aggregation induced by irreversible ADP (10 *μ*M) and collagen (10 *μ*g/mL) stimuli [93]. Apart from single bioactive component, studies have also provided evidences for antiplatelet aggregation effects of crude extracts of natural products. The 80% aqueous-ethanol extract of* Abies webbiana* was found to inhibit both ADP- and epinephrine-induced human platelets aggregation, thereby suggesting therapeutic potential of this plant against thromboembolic conditions [94]. In Gadi's study, crude aqueous extract (CAE) of parsley was evaluated for its antiplatelet aggregation activity in rats* in vitro* and* ex vivo*. CAE dose-dependently inhibited platelet aggregation* in vitro *induced by THR, ADP, collagen and epinephrine. The oral administration of CAE (3 g/kg) significantly (*P* < 0.001) inhibited platelet aggregation* ex vivo* and prolonged bleeding time (*P* < 0.001) without changes of the platelet amount [95]. In terms of the mechanisms for antiplatelet therapies, they are mainly composed of platelet membrane protein inhibitors, impacting nucleotide and arachidonic acid system as well as inhibition of platelet granules secretion.

#### 3.2.1. Inhibition of Platelet Membrane Receptors

Development of definite platelet receptor inhibitors contributed to clinical treatment of antiplatelet aggregation, for example, ADP P2Y_12_ receptor antagonists include ticlopidine and clopidogrel; GPIIb/IIIa antagonists include abciximab, tirofiban, and eptifibatide [96]. Based on the variety of protein structures, functions and ligand properties, platelet receptors can be classified into three groups include integrin, adhesion and agonist receptors. A large number of natural products and their constituents are reported as platelet receptors antagonists (Table 2).

GPIIb/IIIa, a heterodimeric receptor of the integrin family expressed at high density (50000–80000 copies/cell) on the platelet membrane, determines the final process during platelet aggregation. So many new antiplatelet aggregation drugs mainly focus on inhibition of this dominant receptor [151].* Spatholobus suberectus* is a widely used TCM to promote blood circulation for the treatment of diseases related to the blood stasis syndromes [152]. It has been demonstrated that 95% ethanol extract of* S. suberectus* significantly inhibited ADP- and collagen-induced platelet aggregation in human platelet by inhibiting fibrinogen binding to the GPIIb/IIIa receptor and further suppressing the formation of TXA_2_ [106]. Garlic is a common used spicy food all over the world, and a garlic preparation aged garlic extract (AGE) is reported to have inhibition effect of platelet aggregation [153]. Allison et al. [113] investigated the antiplatelet aggregation mechanism of AGE by testing their adhesion to fibrinogen using Rose Bengal and ^51^Cr uptake, fluorescence activated cell sorting (FACS) analysis and measurement of intracellular cAMP contents in human platelet after induced by ADP. The results showed that AGE at concentrations of 3.12% to 12.5% (v/v) can inhibit the binding of platelets to fibrinogen by approximately 40% in the Rose Bengal assay (*P* < 0.05) as well as 61.5%~72% in the ^51^Cr experiments (*P* < 0.05), and significantly decrease the amount of PAC-1 binding to GPIIb/IIIa by approximately 72% in the FACS analysis with increasing platelet cAMP (*P* < 0.01) level. These findings suggested that AGE inhibits platelet aggregation via inhibition of the GPIIb/IIIa receptor and an increase of cAMP level. In Jeon's study, two bioactive compounds isomaltol and pentagalloyl glucose were separated from bark of* Rhus verniciflua *Stokes, and their antiplatelet mechanism were evaluated using receptor expression on platelet membranes, including GPIIb/IIIa (CD41), GPIIb/IIIa-like expression (PAC-1) and P-selectin (CD62), and intracellular calcium mobilization responses. The results indicated that pentagalloyl glucose had a significant inhibitory effect on the expression of P-selectin, but isomaltol had no such effect. Furthermore, isomaltol and pentagalloyl glucose decreased the expression of GPIIb/IIIa, which appeared to have anti-GPIIb/IIIa activity [118].

Adhesion receptors, which mainly refer to collagen receptors, mediate the platelet binding to injury endothelium including *α*_2_ *β*_1_ (GPIa/IIa) and GPVI. Glaucocalyxin A (GLA) is a biologically active ent-kauranoid diterpenoid isolated from* Rabdosia japonica* var.* glaucocalyx*, a traditional Chinese medicinal herb. GLA can significantly inhibit platelet aggregation in response to most of the platelet agonists including collagen, THR and ADP [154]. The inhibitory effect of GLA on collagen-stimulated platelet aggregation was notably potent, even occurred at as low as 0.01 *μ*g/mL. GLA inhibited platelet aggregation induced by collagen-related peptide (CRP), a GPVI specific agonist in a dose-dependent manner and reduced collagen-induced phosphorylation of three major molecules, tyrosine kinase Syk, LAT, and phospholipase C*γ*2 in GPVI signaling pathway. Therefore, GLA can be developed and used as an collagen receptor antagonist for antiplatelet aggregation [108]. Salvianolic acid B (SB) is an active component isolated from Danshen (*Salvia miltiorrhiza*), a TCM widely used for the treatment of cardiovascular disorders. Ma et al. demonstrated that *α*_2_ *β*_1_ might be one of the direct target proteins of SB on platelets, and the signal cascade network of SB after binding with integrin *α*_2_ *β*_1_ might include regulation of intracellular Ca^2+^ level, cytoskeleton-related proteins such as coronin-1B and cytoskeleton structure of platelets [109]. A traditional Korean formula called modified Je-Ho-Tang (MJHT), which is composed of Mume Fructus, Amomi Tsaoko Fructus, Santali Albi Lignum and Amomi Fructus, could promote blood flow and eliminate blood stasis. The hot-water extract of MJHT dose-dependently inhibited collagen-induced whole blood aggregation and adhesion by shear stress in flow conditions. Besides, the extract significantly inhibited the conformational change of GPIIb/IIIa (PAC-1), the activation of P-selectin and mobilization of platelet Ca^2+^ [120].

Once adhere to the sites of vascular injury, platelets are involved in the process of activation and aggregation by releasing of agonists such as ADP, 5-HT, TXB_2_ to amplify the thrombus. Therefore, inhibition of the agonist' receptor can attenuate the formation of thrombus. Two active components, acacetin and II-3,I-5,II-5,II-7,I-4′,II-4′-hexahydroxy-(I-3,II-8)-flavonylflavanonol from the leaves of* Garcinia nervosa* var.* pubescens* King, showed strong inhibitory effects on platelet-activating factor (PAF) receptor [111]. Another agonist receptor of THR could be strongly inhibited by Eryloside F, a novel steroidal disaccharide metabolite of* Erylus formosus*, and finally leaded to inhibit human platelet aggregation* in vitro* [117].* Piper longum* L. has been used as a crude drug to improve intestinal disorder as well as the activity of peripherally poor blood circulation in Asia [155]. Piperlongumine, a constituent of* P. longum*, could concentration-dependently inhibited platelet aggregation induced by TXA_2_ receptor agonist U46619, but slightly inhibited THR-induced aggregation. Piperlongumine also inhibited U46619-induced phosphatidylinositol hydrolysis and the binding of (^3^H)SQ29548 (TXA_2_ receptor antagonist) to TXA_2_ receptor, so it is assumed that piperlongumine act as a TXA_2_ receptor antagonist to inhibit platelet aggregation [119]. Pomolic acid (PA), triterpenoid isolated from* Licania pittieri*, has shown a potent ability to inhibit ADP- and epinephrine-induced human platelet aggregation. According to the mechanism study, PA could be a potent competitive antagonist of P2Y_12_ receptor [121].

#### 3.2.2. Impacting on Nucleotide System

cAMP plays a modulatory role in PLC-mediated secretion and aggregation of human platelets. The levels of cAMP are tightly controlled and dependent on both its synthesis rate by adenylate cyclase (AC) and its hydrolysis rate by PDE [156]. In addition, cAMP levels may be increased by peroxisome proliferator-activated receptors (PPARs) activation [157]. Intracellular cyclic guanosine monophosphate (cGMP) levels are rapidly increased by soluble guanylyl cyclase (sGC), which modulates multiple signaling pathways, including cGMP-dependent receptor proteins, cGMP-regulated PDE and cGMP-dependent protein kinases. The increasing in cGMP levels is accompanied by a decrease in intracellular Ca^2+^ mobilization while the decrease in Ca^2+^ levels inhibits the conformation change of GPIIb/IIIa into its active form and thus decreases platelet binding to fibrinogen [158]. In a word, the increasing in cAMP and cGMP levels may exert a strong platelet inhibitory effect by decrease of intracellular Ca^2+^ levels.

Cordycepin (3′-deoxyadenosine), the major active component in* Cordyceps militaris*, had significant inhibition effect on human platelet aggregation. Cordycepin may increase cAMP and cGMP levels and subsequently inhibit the intracellular Ca^2+^ as well as TXA_2_ but without affecting on PLC-*γ*2 or IP3 [159]. In another study, cordycepin-enriched- (CE-) WIB801C from* Cordyceps militaris *dose-dependently inhibited ADP-induced platelet aggregation with IC_50_ of 18.5 *μ*g/mL. The possible inhibition mechanism was that CE-WIB801C elevated cAMP involved in IP_3_RI (Ser^1756^) phosphorylation to inhibit Ca^2+^ mobilization and VASP (Ser^157^) phosphorylation to inhibit *α*_IIb_/*β*_3_ activation [160]. The ancient plant* Ginkgo biloba* possesses many biological activities such as radical scavenging, blood flow improvement and vasoprotection. Ginkgolide C, one of the active components in* G. biloba*, can significantly increase the formation of cAMP and cGMP as well as suppressing the level of intracellular Ca^2+^ and TXA_2_. In addition, zymographic analysis confirmed that pro-matrix metalloproteinase-9 (pro-MMP-9, 92-kDa) released from human platelets can be activated by Ginkgolide C to form an activated MMP-9 (86-kDa), which can significantly inhibit platelet aggregation stimulated by collagen [161]. Furthermore, another active component of* G. biloba*, quercetin prevented platelet aggregation by inhibition of PDE_3_ [162]. It should be mentioned that PDEs can limit the intracellular levels of cyclic nucleotides by catalyzing the hydrolysis of cAMP and cGMP, thus regulating platelet function. The inhibition of PDEs may therefore exert a strong platelet inhibitory effect [163]. Oligoporin A from* Oligoporus tephroleucus*, an edible mushroom cultivated in Korea, inhibited collagen-induced platelet aggregation in a concentration-dependent manner, but not affecting ADP- and THR-induced platelet aggregation. Further study revealed that oligoporin A can induce the dynamic increase of cAMP and cGMP in platelet. Rat blood* in vitro *pretreatment with oligoporin A significantly blocked collagen-induced ERK2 phosphorylation as well as diminished the binding of fibrinogen to its cognate receptor, integrin *α*_IIb_/*β*_3_ [164].

#### 3.2.3. Inhibition of Platelet Granules Secretion

Platelet granules mainly consist of *α*-granules, dense granules and lysosomes which serve an essential role in promoting platelet aggregation by releasing numerous activated factors such as Ca^2+^, 5-HT, ATP, ADP, P-selectin, and so forth [165]. Inhibitions of platelet granules secretion by natural products are summarized in Table 3.

The concentration of cytosolic Ca^2+^ plays a fundamental role in mediating dense granule release and platelet aggregation. Crocetin, a major ingredient of saffron, against platelet aggregation were mainly contributed to inhibiting Ca^2+^ mobilization via reducing both intracellular Ca^2+^ release and extracellular Ca^2+^ influx, as well as inhibiting secretion of 5-HT, an independent risk factor for platelet aggregation and for thrombus formation [122]. Geiji-Bokryung-Hwan (GBH), Korean traditional formulation, consisting of Cinnamomi Ramulus, Poria Cocos, Mountan Cortex Radicis, Paeoniae Radix and Persicae Semen. GBH potently inhibited thrombin, CRP, U46619 (a TXA_2_ mimic), ADP, or SFLLRN (a thrombin receptor agonist peptide) induced platelet aggregation by acting on a certain step of the signal transduction pathway. Park et al. confirmed that GBH inhibited IP3-mediated Ca^2+^ mobilization without altering tyrosine phosphorylation of PLC-*γ*2 [124]. Magnolol was isolated from Magnolia bark for the treatment of anxiety, neural and cardiovascular disorders [166], the antiplatelet aggregation mechanism of magnolol contribute to an inhibitory effect on 5-HT releasing [126]. Curdione, one of the major sesquiterpene compounds from Rhizoma Curcumae, had a potent protective effect on acute liver injury in mice and potentially to be an active constituent for strengthening the anti-inflammatory or cancer chemo-preventive capacity [167]. In the antiplatelet aggregation test, curdione preferentially inhibited PAF- and THR-induced platelet aggregation in a concentration-dependent manner (IC_50_ = 60–80 *μ*M). Curdione can inhibit P-selectin expression, intracellular Ca^2+^ mobilization as well as causing an increase of cAMP levels in PAF-activated platelets [132].

P-selectin, shows a crucial function in mediating platelet adhesion to the damage vessels, is localized in the *α*-granules and released when activation of platelet. Black soybean (BB) significantly inhibited collagen-induced platelet aggregation by attenuating 5-HT secretion and P-selectin expression, as well as inhibiting TXA_2_ formation* in vitro* [125]. Ligustrazine ferulate, the main active component of* Rhizoma Ligustici Chuanxiong* had distinct antithrombotic effect. Ligustrazine ferulate reduced the expression of platelet P-selectin as well as suppression of platelet adhesion to neutrophil [127]. Soshiho-tang (SH), which consists of seven herbal drugs, had antithrombotic and antiplatelet activities. Lee et al. reported that SH significantly inhibited various agonist-induced platelet aggregations and completely inhibited 5-HT secretion and TXA_2_ formation. Furthermore, SH presented antithrombotic activity by prolonging the occlusion time of thrombus formation when applied in a FeCl_3_-induced thrombus formation model [123]. Fuentes et al. demonstrated for the first time that guanosine from* Solanum lycopersicom* possessed antiplatelet (secretion, spreading, adhesion and aggregation) activity induced by ADP as well as collagen* in vitro* and inhibited platelet inflammatory mediator of atherosclerosis (sCD40L), while depression of CD40L expression can prevent thromboembolic-related disorders [131].

#### 3.2.4. Impacting on Arachidonic Acid System

TXA_2_, intensely induces platelet activation and vasoconstriction, is generated from arachidonic acid (AA) which released when membrane phospholipids are broken down by diverse agonists such as collagen, thrombin and ADP. The enzymes related to TXA_2_ production are cyclooxygenase (COX-1) and thromboxane synthase (TXAS), which are located at microsomes. COX-1 produces prostaglandin (PGG_2_) from substrate AA, TXAS produces TXA_2_ from PGH_2_ that oxidized from PGG_2_ by endoperoxidase. Therefore, inhibition of COX-1 or TXAS is a very useful marker to evaluate the antiplatelet effect of compound. For instance, COX-1 inhibitor aspirin and TXAS inhibitor ozagrel are being used as antiplatelet agents [168]. Another metabolic pathway of AA is the lipoxygenase (LOX) pathway that forms hydroxyeicosatetraenoic acids (HETE) and leukotrienes. TXB_2_ and 6-keto-PGF1_*α*_ are the stable metabolites of TXA_2_ and PGI_2_, respectively. When the ratio of TXA_2_/PGI_2_ is above normal conditions, thrombus formation will occur. On the other hand, when the ratio of TXA_2_/PGI_2_ is lower than normal conditions, the processes of platelet aggregation or thrombus formation will be self-limited and a bleeding tendency may occur. A variety of natural products (Table 4) including berberine [138], hesperetin [139] and ethyl acetate extract of* Caesalpinia sappan* L. [145] inhibited platelet aggregation by keeping balance of TXA_2_ and PGI_2_.

As mentioned above, interference of the activation of the associated enzymes such as COX-1, COX-2, TXAS and LOX during arachidonic acid pathway is regarded as an effective way to inhibit platelet aggregation. Obovatol, a major biphenolic component of* Magnolia obovata* leaves, presented antiplatelet activity by inhibiting COX-1 and LOX activities to suppress production of TXB_2_, PGD_2_ and 12-HETE [136]. Morroniside, extracted and purified from* Cornus officinalis* Sieb.et Zucc, significantly inhibited the activation of COX as well as TXB_2_ generation, and had a selective antiplatelet effect on ADP-induced aggregation [146, 147]. Coy et al. isolated 26 neolignans (14 bicyclooctane-type and 12 benzofuran-type) from three Lauraceae species (*Pleurothyrium cinereum*,* Ocotea macrophylla*, and* Nectandra amazonum*) and evaluated their antiplatelet aggregation property* in vitro *through inhibition of COX-1, COX-2, 5-LOX and agonist-induced aggregation of rabbit platelets. The results showed that benzofuran neolignans were found to be the COX-2 selective inhibitors, whereas bicyclooctane neolignans selectively inhibited the PAF-action as well as COX-1 and 5-LOX. The neolignan 9-nor-7, 8-dehydro-isolicarin B, and cinerin C were found to be the most potent COX-2 inhibitor and PAF-antagonist, respectively. In addition, nectamazin C (bicyclooctane-type neolignan) exhibited dual 5-LOX/COX-2 inhibition [148]. Abe et al. screened for inhibitors of human platelet aggregation and human 5-LOX from the Myoga (*Zingiber mioga* Roscoe) extracts. Experimental results indicated that miogatrial, miogadial, sesquiterpene and polygodial were potent inhibitors of human platelet aggregation and human 5-LOX, and their 3-formyl-3-butenal structure was essential for the activities [149]. In addition, Ginsenoside Rk1 from white ginseng decreased the 12-HETE level involved in AA pathway, which is related to 12-LOX translocation resulting from the decreased of Ca^2+^ levels [150].

### 3.3. Fibrinolysis

The conversion of fibrinogen to fibrin and the consequent formation of a stable fibrin clot are the ultimate events in the coagulation and thrombotic cascades [169]. The agents available for clinical treatment on fibrinolysis can be classified into two groups: plasmin-like proteases which can directly hydrolyse fibrin, for example, nattokinase and lumbrokinase; and plasminogen activators, for example, tissue type plasminogen activator (t-PA) and streptokinase [170]. In recent years, some effective thrombolytic agents have been purified and characterized from foods or animal materials such as Japanese natto, douche (a traditional Chinese soybean food) [171] and earthworm [172].

In 1983, a high fibrinolytic active enzyme named lumbrokinase was firstly separated from artificial breeding earthworm in Japan [173]. This fibrinolytic enzyme had a dual functions included dissolving fibrin directly and activate plasminogen. Furthermore, Mihara et al. [172] isolated a strong fibrinolytic enzyme from* Lumbricus rubeulls* which contained abundant asparagine and aspartic acid with little proline or lysine. In addition, Xiong et al. separated and purified a fibrinolytic enzyme (33 kDa) with strong fibrinolysis effects and proteolytical activity from* Eisenia foelide* [174].

Nattokinase (27.3 kDa to 35 kDa) is a kind of serine proteases which is produced in the fermentation process of* Bscillus natto* or* Bacillus subtilis *var.* natto*. Nattokinase possesses a significant fibrinolytic property and the main mechanisms were to dissolving fibrin directly as well as activating plasminogen to increase the intrinsic plasmin formation. In the expectation to be developed as a new generation of fibrinolytic agents and health food, nattokinase has lots of advantages such as high safety, low cost and fast acting [175, 176]. Another serine protease (31 kDa with a single polypeptide chain) with fibrinolytic activity named CSP was purified from the culture supernatant of the fungus* Cordyceps sinensis*. CSP was found to be a plasmin-like protease, but not a plasminogen activator through preferentially cleaving the A*α* chain of fibrinogen and the *α*-chain of fibrin [170].

*Pinus densiflora*, an evergreen needle-leafed tree indigenous to Asia Pacific, has been used for the treatment of multiple ailments such as cardiovascular disease, cancer, diabetes and antihypertension. It was reported that pine needle extract would facilitate fibrinolysis, decrease the blood plasma cholesterol and triglyceride in cholesterol fed rat, and it's helpful in removing blood clots [177]. On the other hand, Huang et al. screened for the fibrinolytic activities of 6 kinds of authentic medicinal materials from Guangxi (China) by fibrin plate method* in vitro*. As a result,* Pueraria lobata*,* Trichosanthes kirilowii*,* Lonicera japonica*, and* Desmodium styracifolium* showed fibrinolytic activity, and in particular the fibrinolytic activity of* D. styracifolium* was similar to that of positive drug urokinase [178]. In addition, two components (1-palmitoyl-2-oleoyl-3-*O*-*α*-D-glucopyranosylglycerol and 1-myristoyl-2-oleoyl-3-*O*-*α*-D-glucopyranosylglycerol) were purified from* Sargassum fulvellum* and the fibrinolytic effect was identified* in vitro* [179].

## 4. Conclusion

Thrombosis remains a final pathway to disease and death in some of our most common diseases such as myocardial infarction and stroke. Although substantial progress has been made in understanding the biology of thrombus formation and the pathophysiology of thrombosis, all the pharmacological agents available for prevention or treatment have been in use for decades or have been replaced with newer variants that offer a modest incremental improvement. Natural products have been reported with apparent inhibitory activity on thrombotic diseases both in experimental and clinical stages, which provide a useful preventive approach or an adjunct to current pharmacological treatments for thrombotic diseases. Advances in the knowledge of both the mechanisms of thrombus formation and of the biological functions of natural products will provide new insights to promote human health.

## Figures and Tables

**Table 1:** Inhibition on the coagulation pathways of natural products.

Natural products	Experimental models	Pathways	Effects	Reference
Polysaccharide HAF0 of *Monostroma arcticum *	Human blood (*in vitro*)	IN & CO	Prolonging APTT and TT, but without PT	[80]

Total saponin of *Polygala fallax* Hesml.	Rabbit blood (*in vitro*)	IN	Prolonging APTT and RT and fibrinogen clotting time, but without PT	[81]

Borneol	Rat blood (*ex vivo*)	EX & CO	Prolonging PT and TT and inhibition of arteriovenous shunt as well as venous thrombosis	[97]

Withaferin A of *Withania somnifera *	Human blood (*ex vivo*)	IN & CO	Prolonging APTT and PT and inhibition of thrombin, FXa formation, and TNF-*α* induced PAI-1 production as well as extending *in vivo* and *ex vivo* bleeding time	[86]

Saline extract of *Hirudinaria manillensis *	Rat blood (*ex vivo*)	IN, EX & CO	Prolonging APTT, PT, and TT	[98]

Total glycosides of paeony	Rabbit blood (*in vitro*)	IN, EX & CO	Prolonging APTT, TT, and PT	[90]

95% ethanol extract of *Ferula lehmannii* Boiss.	Rat blood (*ex vivo*)	IN, EX & CO	Prolonging APTT, TT, and PT	[99]

Dilinoleic acid, safflower yellow, and compatibility preparation	Rat blood (*ex vivo*)	IN & EX	Prolonging APTT, TT, CT, and BT	[100]

Aqueous extract of *Whitmania pigra* Whitman	Rat blood (*in vitro*)	IN & EX	Prolonging APTT as well as TT and suppression of fibrinogen formation	[101]

Phlorotannins STP-1 and STP-2 of *Sargassum thunbergii* Kuntze	Rabbit blood (*in vitro*)	IN, EX & CO	Prolonging APTT, TT, PT, CT, and BT	[102]

Sulfated polysaccharides of *Hizikia fusiformis *	Rat/Rabbit blood (*in vivo/in vitro*)	IN	Prolonging rats BT, CT *in vivo,* and rabbits APTT* in vitro *	[103]

Hyperoside of *Rhododendron brachycarpum *	Rat blood (e*x vivo*)	IN & EX	Prolonging APTT and PT	[82]

Polysaccharide of *Umbilicaria esculenta *	Rat blood (i*n vitro*)	IN, EX & CO	Prolonging APTT, PT, and TT	[83]

Sulfated (1→3)-*β*-L-arabinan of *Codium vermilara *	Human blood (i*n vitro*)	IN, EX & CO	Prolonging APTT, PT, and TT	[104]

Wogonin and wogonoside of* Scutellaria baicalensis *Georgi	Human blood (*in vitro*)	IN & EX	Prolonging APTT and PT and inhibition of the activities and production of THR and FXa	[88]

Crude extracts of *Erigeron canadensis *L.	Human blood (i*n vitro*)	IN & EX	Prolonging APTT and PT	[89]

IN, EX, and CO represent for intrinsic, extrinsic, and common coagulation pathways, respectively; APTT: activated partial thromboplastin time; TT: thrombin time; PT: prothrombin time; RT: recalcification time; CT: coagulative time; BT: bleeding time.

**Table 2:** Inhibition of platelet membrane receptors of natural products.

Natural products	Experimental models	Possible mechanisms	Reference
2,3,5,4′-Tetrahydroxystilbene-2-O-*β*-D-glucoside of *Polygonum multiflorum *	Human blood (*in vitro*); agonist: collagen	Inhibition of Fc*γ*RIIa, Akt (Ser473), and GSK3*β* (Ser9) phosphorylation	[105]

95% ethanol extract of *Spatholobus suberectus *	Human blood (*in vitro*); agonist: collagen	Blockage of fibrinogen binding to the GP IIb/IIIa, suppression of TXA_2_ formation	[106]

A new tripeptide (AAP) of *Agkistrodon acutus* Venom	Rabbit blood (*in vitro*); agonist: ADP, PAF-acether, collagen and THR	Inhibition of fibrinogen binding to GP IIb/IIIa	[107]

Glaucocalyxin A of *Rabdosia japonica* (Burm. f.) var. *glaucocalyx* (Maxim.) Hara	Human blood (*in vitro*); agonist: collagen	Inhibition of tyrosine phosphorylation of Syk, LAT, phospholipase C*γ*2, and P-selectin secretion	[108]

Salvianolic acid B of *Salvia miltiorrhiza *	Rat blood (*in vitro and ex vivo*); agonist: collagen	Exerting binding affinity to *α*_2_ *β*_1_, decreasing of intracellular Ca^2+^, and impacting on cytoskeleton-related proteins level	[109]

Indole-3-carbinol of cruciferous vegetables	Human blood (*in vitro*); agonist: collagen	Inhibition of fibrinogen binding to GP IIb/IIIa and decreasing the levels of TXB_2_, prostaglandin E_2_	[110]

II-3,I-5,II-5,II-7,I-4′,II-4′-Hexahydroxy-(I-3,II-8)-flavonylflavanonol and acacetin of *Garcinia nervosa *var. *pubescens* King	Rabbit blood (*in vitro*); agonist: PAF	Possessing strong PAF antagonistic activity	[111]

Essential oils of five *Goniothalamus* species	Human blood (*in vitr*o); agonist: ADP, AA, and collagen	Possessing strong PAF antagonistic activity	[112]

15–20% ethanol extract of aged garlic	Human blood (*in vitro*); agonist: ADP	Inhibition of fibrinogen binding to GP IIb/IIIa and increasing the level of cAMP	[113]

Tetramethylpyrazine of *Ligusticam wallichii *Franch	Human blood (*in vitro*); agonist: ADP, collagen, and U46619	Inhibition of fibrinogen binding to GP IIb/IIIa and the levels of intracellular Ca^2+^ as well as TXB_2_	[114]

Aqueous extract of *Agrimonia pilosa *	Human blood (*in vitro*); agonist: ADP	Inhibition of fibrinogen binding to GP IIb/IIIa and decreasing the level of P-selectin	[115]

N-butanol extract of *Toona sinensis* Seed	Human blood (*in vitro*); agonist: THR	Inhibition of fibrinogen binding to GP IIb/IIIa and decreasing the level of intracellular Ca^2+^	[116]

Eryloside F of Erylus formosus	Human blood (*in vitro*); agonist: THR, SFLLRN, and U-46619	Possessing strong THR antagonistic activity	[117]

Isomaltol and pentagalloyl glucose of* Rhus verniciflua* Stokes	Human blood (*in vitro*); agonist: ADP, AA, and collagen	Decreasing the expression of GPIIb/IIIa	[118]

Piperlongumine of *Piper longum* L.	Rabbit blood (*in vitro*); agonist: U4619 and THR	Inhibition of U46619-induced phosphatidylinositol hydrolysis as well as the binding of (^3^H)SQ29548 to TXA_2_ receptor	[119]

Hot-water extract of modified Je-Ho-Tang (Mume Fructus, Amomi Tsaoko Fructus, Santali Albi Lignum, and Amomi Fructus)	Human blood (*in vitro*); agonist: collagen	Inhibiting adhesion and decreasing the activation of GPIIb/IIIa-like expression and P-selectin monoclonal, Ca^2+^ mobilization	[120]

Pomolic acid of *Licania pittieri *	Human blood (*in vitro*); agonist: ADP	Competitive antagonism of ADP-induced platelet aggregation	[121]

ADP: adenosine diphosphat; PAF: platelet activating factor; THR: thrombin; AA: arachidonic acid; SFLLRN: thrombin receptor activating peptide; GP IIb/IIIa: Glycoprotein IIb/IIIa; TXA_2_: thromboxane A_2_; TXB_2_: thromboxane B_2_; cAMP: cyclic adenosine monophosphate; (^3^H)SQ29548: TXA_2_ receptor antagonist.

**Table 3:** Inhibition of the platelet granules secretions of natural products.

Natural products	Experimental models	Possible mechanisms	Reference
Crocetin of Saffron	Rat blood (*ex vivo*); agonist: ADP	Inhibition of Ca^2+^ mobilization via reducing both intracellular Ca^2+^ release and extracellular Ca^2+^ influx as well as 5-HT secretion	[122]

Aqueous extract of Soshiho-tang	Rat blood (*in vitro*); agonist: collagen, THR and AA	Inhibition of 5-HT and TXA_2_ formation	[123]

Geiji-Bokryung-Hwan (*Cinnamomi Ramulus, Poria Cocos, Mountan Cortex Radicis, Paeoniae Radix, *and* Persicae Semen*)	Human blood (*in vitro*); agonist: THR and CRP	Inhibition of IP3-mediated Ca^2+^ mobilization	[124]

20% ethanol extract of black soybean	Human blood (*in vitro*); agonist: collagen	Attenuating 5-HT secretion and P-selectin expression, and inhibiting TXA_2_ formation	[125]

Magnolol of magnolia bark	Rabbit blood (*in vitro*); agonist: collagen	Inhibition of 5-HT secretion	[126]

Ligustrazine ferulate of Rhizoma Ligustici Chuanxiong	Rat blood (*ex vivo*); agonist: THR	Reduction of the expression of platelet P-selectin as well as suppression of platelet adhesion to neutrophil	[127]

Dihydroxybenzyl alcohol of *Gastrodia elata *Blume.	Rabbit blood (*in vitro*); agonist: AA	Inhibition of Ca^2+^ mobilization via reducing both intracellular Ca^2+^ release and extracellular Ca^2+^ influx	[128]

Rhynchophylline	Rabbit blood (*in vitr*o); agonist: ADP and THR	Inhibition of Ca^2+^ mobilization via extracellular Ca^2+^influx rather than intracellular Ca^2+^ release	[129]

Salvianolic acid B of *Salvia miltiorrhiza *	Human blood (*in vitro*); agonist: ADP and THR	Inhibition of P-selectin and CD40L releasing	[130]

Guanosine of *Solanum lycopersicum *	Human blood (*in vitro*) agonist: ADP and collagen	Inhibition of CD40L and ATP secretion	[131]

Curdione of Rhizoma Curcumae	Human blood (*in vitro*) agonist: THR, PAF, ADP and AA	Inhibition of P-selectin expression, intracellular Ca^2+^mobilization and increasing the cAMP levels in PAF-activated platelets	[132]

ADP: adenosine diphosphate; THR: thrombin; AA: arachidonic acid; CRP: collagen-related peptide; 5-HT: 5-hydroxytryptamine; IP3: inositol-1,4,5-trisphosphate; TXA_2_: thromboxane A_2_.

**Table 4:** Impacting on the arachidonic acid system of natural products.

Natural products	Experimental models	Possible mechanisms	Reference
Epigallocatechin-3-gallate of green tea leaves	Rat blood (*in vitro*); agonist: collagen	Inhibiting the activation of COX-1 and TXAS, with a stronger selectivity in COX-1 inhibition than TXAS inhibition	[133]

Jujuboside B of seeds of *Zizyphus jujuba *	Rat blood (*in vitro*); agonist: collagen	Inhibition of TXA_2_ production	[134]

Alditol and monosaccharide of sorghum vinegar	Human blood (*in vitro*); agonist: AA, collagen, ADP, and THR	Inhibition of COX-1 and TXAS and attenuating TXA_2_ production	[135]

Diacetylated obovatol of *Magnolia obovata *leaves	Rabbit blood (*in vitro*); agonist: collagen and AA	Inhibition of COX-1 and LOX activities and decreasing in cytosolic Ca^2+^ mobilization and 5-HT secretion	[136]

Ethanol extract, eupatilin, and jaceosidin of *Artemisia princeps* Pampanini	Human blood (*in vitro*); agonist: AA	Inhibition the generation of 5-HT and TXA_2_	[137]

Berberine of berberine sulfate injection	Rabbit blood (*ex vivo*); agonist: ADP, AA, and collagen	Suppressing of TXA_2_	[138]

Hesperetin of grapefruits and oranges	Rabbit blood (*in vitro*); agonist: AA and collagen	Inhibition of PLC-*γ*2 phosphorylation, COX-1 activity, and decreasing of Ca^2+^ as well as TXA_2_	[139]

Green tea catechins of *Camellia sinensis *	Rabbit blood (*in vitr*o); agonist: AA, collagen, and U-46619	Inhibition of AA liberation, TXA_2_ synthesis, PGD_2_, and ATP formation	[140]

Hydroxychavicol of betel quid	Rat blood (*in vitro*); agonist: AA, collagen, and THR	Inhibition of COX-1/COX-2 enzyme activity and decreasing TXA_2 _and ROS production as well as Ca^2+^ mobilization	[141]

Tetrandrine and fangchinoline of *Radix Stephaniae Tetrandrae *	Human blood (*in vitro*); agonist: PAF, THR and AA	Suppression of TXA_2_ formation, but without inhibiting the binding of PAF to PAF-receptor	[142]

Isorhynchophy lline of *Uncaria sinensis* (Oliv.) Havil.	Rabbit blood (*in vitro*); agonist: collagen	Inhibition of TXA_2_ formation	[143]

Genistein	Rabbit blood (*in vitro*); agonist: PAF	Inhibition of TXA_2_ formation and increasing PGI_2_ generation	[144]

Ethyl acetate extract of *Caesalpinia sappan* L.	Rat blood (*ex vivo*); agonist: ADP	Inhibition of TXA_2_ formation and increasing PGI_2_ generation	[145]

Morroniside of *Cornus officinalis *Sieb.et Zucc	Rabbit blood (*in vitro*); agonist: ADP	Inhibition of COX activation and decreasing TXB_2_ generation	[146, 147]

Neolignans of three Lauraceae species (*Pleurothyrium cinereum, Ocotea macrophylla, and Nectandra amazonum*)	Rabbit blood (*in vitro*) agonist: PAF, ADP and AA	Inhibition of COX-2 by Benzofuran neolignans; inhibition of PAF-action, COX-1, 5-LOX by bicyclooctane; inhibition of COX-2, PAF-action by neolignan 9-nor-7,8-dehydro-isolicarin B and cinerin C; inhibition of 5-LOX/COX-2 by Nectamazin C	[148]

Extracts of Myoga (*Zingiber mioga* Roscoe)	Human blood (*in vitro*) agonist: ADP and AA	Inhibition of 5-LOX by miogatrial, miogadial, sesquiterpene and polygodial	[149]

Ginsenoside Rk1 of white ginseng	Rat blood (*in vitro*) agonist: AA	Decreasing of 12-HETE, 12-LOX, and Ca^2+^ levels	[150]

AA: arachidonic acid; ADP: adenosine diphosphate; THR: thrombin; PAF: platelet activating factor; COX-1: cyclooxygenase-1; COX-2: cyclooxygenase-2; TXAS: thromboxane synthase; LOX: lipoxygenase; TXA_2_: thromboxane A_2_; TXB_2_: thromboxane B_2_; 5-HT: 5-hydroxytryptamine; PLC-*γ*2: phospholipase C-*γ*2; PGD_2_: prostaglandin D_2_; ATP: adenosine triphosphate; ROS: reactive oxygen species; PGI_2_: prostacycline 2; 12-HETE: 12-hydroxy-5,8,10,14-eicosatetraenoic acid.
